# *Lactococcus garvieae* FUA009, a Novel Intestinal Bacterium Capable of Producing the Bioactive Metabolite Urolithin A from Ellagic Acid

**DOI:** 10.3390/foods11172621

**Published:** 2022-08-29

**Authors:** Haoyu Mi, Shu Liu, Yang Hai, Guang Yang, Jing Lu, Fuxiang He, Yaling Zhao, Mengjie Xia, Xiaoyue Hou, Yaowei Fang

**Affiliations:** 1 Co-Innovation Center of Jiangsu Marine Bio-Industry Technology, Jiangsu Ocean University, Lianyungang 222005, China; 2 Jiangsu Key Laboratory of Marine Bioresources and Environment, Jiangsu Ocean University, Lianyungang 222005, China; 3 College of Food Science and Engineering, Jiangsu Ocean University, Lianyungang 222005, China; 4 Key Laboratory of Marine Drugs, The Ministry of Education of China, School of Medicine and Pharmacy, Ocean University of China, Qingdao 266003, China; 5 Jiangsu Marine Resources Development Research Institute, Jiangsu Ocean University, Lianyungang 222005, China; 6 Department of Microbiology, College of Life Sciences, Nankai University, Tianjin 300071, China

**Keywords:** plant-derived activities, *Lactococcus* *garvieae*, ellagic acid, urolithin A, complete genome, safety and probiotic characteristics

## Abstract

Dietary polyphenol ellagic acid has anti-cancer and anti-inflammatory activities, and these biological activities require the conversion of ellagic acid to urolithins by intestinal microbes. However, few gut microbes are capable of metabolizing ellagic acid to produce urolithins, limiting the beneficial effects of ellagic acid on health. Here, we describe an intestinal bacterium *Lactococcus garvieae* FUA009 isolated from the feces of a healthy volunteer. It was demonstrated via HPLC and UPLC-MS analysis that the end product of ellagic acid metabolism of FUA009 was urolithin A. In addition, we also examined the whole genome sequence of FUA009 and then assessed the safety and probiotic properties of FUA009 based on a complete genome and phenotype analysis. We indicated that FUA009 was safe, which was confirmed by FUA009 being sensitive to multiple antibiotics, having no hemolytic activity, and being free of aggressive putative virulence factors. Moreover, 19 stress-responsive protein genes and 8 adhesion-related genes were predicted in the FUA009 genome. Furthermore, we demonstrated that FUA009 was tolerant to acid and bile salt by determining the cell viability in a stress environment. In summary, *Lactococcus* *garvieae* FUA009, as a novel UA-producing bacterium, not only contributes to the study of the metabolic pathway of ellagic acid but is also expected to be a novel probiotic candidate.

## 1. Introduction

Urolithin A (UA), which is a natural metabolite of ellagic acid, was first discovered in 1980 and subsequently detected in other species, such as monogastric animals and humans [1,2]. Many studies have investigated the biological activities of UA. UA could improve heart function in ischemia/reperfusion mice by increasing the antioxidant activity of cardiomyocytes [3]. UA could also act as an autophagy inducer to induce mitochondrial-selective autophagy by activating the PINK1/Parkin ubiquitin-dependent pathway or BNIP3 receptor, thereby reducing the damage to mitochondrial function in aging [4,5]. UA not only could target mitochondrial regulation to maintain energy homeostasis but also has significant curative effects regarding improving chronic inflammation, cardiovascular disease, muscle dysfunction, and neurodegenerative diseases [6,7,8,9]. Moreover, when the model organism *Caenorhabditis elegans* was fed UA for a long time, UA activated mitophagy via the AMPK signaling pathway, extending the lifespan of *C. elegans* by 45.4% compared with the control group [8]. Although UA has not yet been used to assess human lifetime, the results of previous investigations are very promising because UA modulates mitochondrial and cell health in vivo [4]. When California strawberries, which are rich in UA precursors, were added to a diet, both the body weight and gut microorganisms associated with longevity improved in healthy subjects [10]. In addition, UA also plays a significant role in improving human health, such as having positive effects against obesity, intestinal diseases, and cancer [11].

The function of UA is a hotspot in health research, and obtaining UA efficiently and safely has attracted the attention of researchers. Currently, most commercially available UA is obtained using traditional chemical processes [11]. However, chemical methods are not only costly but also pollute the environment. UA does not exist in the natural state but is produced by a series of transformations of ellagitannins (the aggregate state of ellagic acid) by intestinal microbiota [12]. Ellagitannins are natural polyphenol antioxidants that are widely distributed in many fruits (raspberry, blackberry, strawberry, cloudberry, etc.), nuts, and tea [13]. The bioavailability of ellagitannins is very low, and they cannot be completely absorbed into the blood but are hydrolyzed to ellagic acid, which is metabolized by the intestinal microbiota to the more easily absorbed urolithins, such as UA/isourolithin A (dihydroxyurolithin) and urolithin B (monohydroxyurolithin). Moreover, there are several intermediate metabolites (urolithin M5, urolithin D, urolithin M6, urolithin E, urolithin M6R, urolithin C, urolithin CR, urolithin M7R, and urolithin M7) in the ellagic acid metabolic pathway. Urolithin M5 (pentahydroxy-urolithin) is the first urolithin substance produced by the ellagic acid metabolism, and then urolithin M5 is metabolized to tetrahydroxyurolithin (urolithin D, urolithin E, urolithin M6R, and urolithin M6), and trihydroxyurolithin (urolithin C, urolithin CR, urolithin M7R, and urolithin M7) [14]. However, UA is the most biologically active metabolite of all the urolithins, and only 40% of the population can produce UA in vivo and this proportion may also decrease due to the intestinal microbiota change caused by aging [15,16]. According to the type of urolithin metabolism, people are divided into three types: metabotype A, metabotype B, and metabotype 0 [16]. The individuals with metabotype A can metabolize ellagic acid to produce UA, while the individuals with metabotype B produce UA, isourolithin A and/or urolithin B. Metabotype 0 represents the population that cannot produce urolithins. Urolithin metabolism types depend on the composition of human intestinal microbiota [17]. The intestinal microbiota is involved in the regulation of human health through metabolites, such as the ellagic acid metabolite UA.

Intestinal bacteria that are capable of producing the bioactive metabolite UA from ellagic acid are rarely reported. In 2014, the genus *Gordonibacter* (*G. urolithinfaciens* and *G. pamelaeae*) with urolithin-C-producing ability was reported [18,19]. Urolithin C is an intermediate in ellagic acid metabolism. The strain *Ellagibacter isourolithinifaciens* CEBA S4A4, which is capable of producing isourolithin A from ellagic acid, was described in 2017 [20]. At present, only *Bifidobacterium pseudocatemulatun* INIA P815 has been found to produce UA and urolithin B, which was achieved in 2018 [21]. In this study, we isolated a gut bacterium named *Lactococcus garvieae* FUA009 from human feces that is capable of converting ellagic acid to UA under anaerobic fermentation conditions. Moreover, the results of the complete genome and phenotype analysis showed that *Lactococcus garvieae* FUA009 was expected to be developed as a novel probiotic candidate.

## 2. Material and Methods

### 2.1. Chemicals and Solvents

Anaerobic basal broth (ABB) was purchased from Shanghai Ruichu Biotechnology Co., Ltd. (Shanghai, China). Ellagic acid and urolithin A standards were purchased from Yuanye Bio-Technology (Shanghai, China). Urolithins B, C, and M6 were obtained from Standard Technology (Shanghai, China). Urolithins D and isourolithins A were purchased from Toronto Research Chemicals (Toronto, Canada). Methanol, acetic acid, and acetonitrile were obtained from Shanghai Aladdin Biochemical Technology (Shanghai, China).

### 2.2. Isolation of UA-Producing Bacteria from Intestinal Microbiota

Stool samples were obtained from a healthy male donor (aged 26) who was identified as the urolithin A producer in this investigation. Fecal samples from the donor who ate walnuts for a month were diluted 10-fold with sterile saline within one hour of donation. The 1.0 mL diluted sample was added to a 50 mL ABB liquid medium and cultured at 37 °C for 48 h under anaerobic conditions. Then, more than 50 microorganism colonies were obtained from the cell suspensions using plate-screening techniques. Microorganism colonies were found in the ABB solid medium (ABB liquid medium containing 2% agar). Each colony was inoculated separately in ABB liquid medium containing 1% ellagic acid and fermented in anaerobic conditions at 37 °C. Whether the colony could utilize ellagic acid to produce UA was preliminarily analyzed at 0 h, 12 h, 24 h, 36 h, and 48 h using HPLC, and then the UPLC-MS was used to determine whether the metabolite was UA. The conditions for HPLC and UPLC-MS are elaborated below.

### 2.3. HPLC and UPLC-MS Analysis

Samples (1 mL) were collected and extracted with a mixture (C_2_H_3_N:H_2_O:H-COOH) in a volume ratio of 80:19.9:0.1. Then, the extracts were filtered through a 0.22 μm cellulose acetate filter and analyzed using HPLC. HPLC analysis conditions were as follows: the analysis was performed on the Agilent 1260 system using a C18 column (ZORBAX SB-C18, 4.6 mm × 250 mm, 5 μm, Agilent Technologies, Palo Alto, CA, USA). Acetonitrile and 1% methanol were used as the mobile phases. The flow rate and the injection volume were 1.0 mL/min and 5 μL, respectively. UV chromatograms were taken at 305 nm. The gradient profile was 0~15 min, 0~20% acetonitrile; 15~20 min, 20~70% acetonitrile; 20~21 min, 70~95% acetonitrile; 21~24 min, 95~100% acetonitrile; 24~25 min, 100~20% acetonitrile. The ellagic acid metabolite of the suspected UA-producing bacterium was further analyzed using mass spectra UPLC-MS at 305 nm. UPLC-MS was performed on the Waters UPLC system (Waters Ltd., Milford, MA, USA) using the C18 column (ACQUITY UPLC BEH C18, 2.1 × 50 mm, 1.7 μm, 0.5 mL/min) and an ACQUITY QDa ESIMS scan from 150 to 1000 Da. The mobile phase was 0.2% formic acid and acetonitrile with gradient elution. The flow rate and the injection volume were 0.5 mL/min and 50 μL, respectively. The gradient profile was 0~6 min, 10~100% acetonitrile; 6~7 min, 100~100% acetonitrile; 7~8 min, 100~10% acetonitrile; 8~9 min, 10~10%P acetonitrile.

### 2.4. Identification of the UA-Producing Bacteria

The isolated UA-producing bacterium FUA009 was identified using 16S rRNA gene sequence alignment and phylogenetic tree analysis. The 16S rRNA gene sequence, which was amplified from the genomic DNA of FUA009 using primers 27F and 1492R, was analyzed by Qingke Biotechnology (Qingdao, China). The sequencing data were submitted to NCBI GenBank and compared with public sequences in the EMBL database using the BLAST program (National Center for Biotechnology Information, Bethesda, MD, USA). The phylogenetic analysis was performed with MEGA 7.0 (version 7.0, Sudhir Kumar, AZ, USA).

### 2.5. Whole-Genome Sequencing, Assembly, and Annotation

The whole genome of *Lactococcus garvieae* FUA009 was sequenced using the PacBio Sequel platform and Illumina NovaSeq PE150 at the Beijing Novogene Bioinformatics Technology Co., Ltd (Beijing, China). SMRT Link v5.0.1 (version 5.0.1, Pacific Biosciences of California, Inc, Menlo Park, CA, USA) was used for preliminary assembly. Using the variant Caller module of the SMRT Link software, the arrow algorithm was used to correct and count the variant sites in the initial assembly results.

The corrected assembly result, which was used as the reference sequence, was a BLAST with Illumina data using bwa. Furthermore, the result was filtered with a base minimum mass value of 20, a minimum read depth of 4, and a maximum read depth of 1000. Based on the overlap between the head and the tail, we confirmed whether the chromosomal sequence formed a circle or not, then corrected the initial site using BLAST with the DNA database. At last, the chromosome and plasmid sequences were screened using BLAST with the plasmid database.

The genome component prediction included the prediction of the coding gene, repetitive sequences, non-coding RNA, genomics islands, prophage, and clustered regularly interspaced short palindromic repeat sequences (CRISPR). We used two databases to predict gene functions: NR (Non-Redundant Protein Database) and Swiss-Prot. A whole-genome BLAST search (E-value less than 1 × 10^−5^, minimal alignment length percentage larger than 40%) was performed against the above two databases. For pathogenic bacteria, we added the pathogenicity and drug resistance analyses. We used the VFDB (Virulence Factors of Pathogenic Bacteria) to perform the above examinations. The draft genome data of *Lactococcus garvieae* FUA009 was finally deposited in GenBank (accession number: PRJNA851534).

### 2.6. Safety Assessment of the UA-Producing Bacterium FUA009

#### 2.6.1. Hemolysis Activity and Antibiotic Susceptibility Assay

The hemolytic potential of *Lactococcus* *garvieae* FUA009 was measured by streaking the cells onto blood agar with 6% sheep blood. *Staphylococcus aureus* served as the positive control. Plates were incubated at 37 °C for 24 h under anaerobic conditions. The antibiotic susceptibility of *Lactococcus* *garvieae* FUA009 was performed using the Kirby–Bauer (KB) disk diffusion technique with the following concentration disks: amikacin 30 μg, norfloxacin 10 μg, ofloxacin 5 μg, ciprofloxacin 5 μg, levofloxacin 5 μg, erythromycin 15 μg, tetracycline 30 μg, cefuroxime 30 μg, cefazolin 30 μg, cefalotin 30 μg, cefotaxime 30 μg, cefatriaxone 30 μg, ceftazidime 30 μg, piperazoline 100 μg, ampicillin 10 μg, oxacillin 1 μg, penicillin G 10 μg, aztreonam 30 μg, co-trimoxazole 23.75 μg, furadantin 300 μg, chloramphenicol 30 μg, bacillosporin B 300 μg, clindamycin 2 μg, kanamycin 30 μg, gentamicin 10 μg, streptomycin 10 μg, and vancomycin 30 μg. The results were interpreted by measuring the inhibition zone diameters and were categorized as sensibility, intermediacy, and resistance in accordance with the Clinical and Laboratory Standards Institute [22]. Each experiment was performed in triplicate.

#### 2.6.2. Genome Mining for the Safety-Related Genes and Mobile Genetic Elements

The putative virulence factor genes and antibiotic resistance genes of *Lactococcus* *garvieae* FUA009 were performed using the Virulence Factor Database (VFDB) and Antibiotic Resistance Genes Database (ARDB), respectively. The capture, accumulation, and dissemination of resistance genes were largely due to the actions of mobile genetic elements (MGEs), such as genomic islands (GIs), prophages, and plasmids [23]. The GIs, prophages, and plasmid elements were predicted with IslandPath-DIOMB, PhiSpy, and the plasmid database, respectively.

### 2.7. Probiotic Characteristics Assessment of the UA-Producing Bacterium Lactococcus garvieae FUA009

#### 2.7.1. Identification of Probiotic Related Genes in the *Lactococcus* *garvieae* FUA009 Genome

Hidden Markov models (HMMs) were used to detect the probiotic-related genes, such as those for acid, bile salt, temperature, metal, and oxidative tolerance. Furthermore, the keywords were searched for in the annotation results of Swiss-Prot and Pfam for the related genes of the adhesion factors.

#### 2.7.2. Evaluation of the Acid and Bile Salt Tolerance In Vitro

The tolerance of *Lactococcus* *garvieae* FUA009 to acid and bile salt was measured using the viable plate count method. Bacterial cell suspensions with an inoculation of 2% were cultured in ABB medium with different pHs (2.0, 2.5, 3.0, 3.5, 4.0) or ABB medium with different bile salts (0.1%, 0.2%, 0.3%, 0.4%, 0.5%), incubated at 37 °C under anaerobic conditions for 0–3 h, and then spread on ABB agar plates. Finally, the number of viable colonies on the plate was counted. The survival rate (SR) was calculated as follows: SR = (N_t_/N_0_) × 100%, where N_t_ and N_0_ represent the experimental group and control group, respectively.

## 3. Results

### 3.1. Isolation and Identification of UA-Producing Bacteria

**Figure 1:**
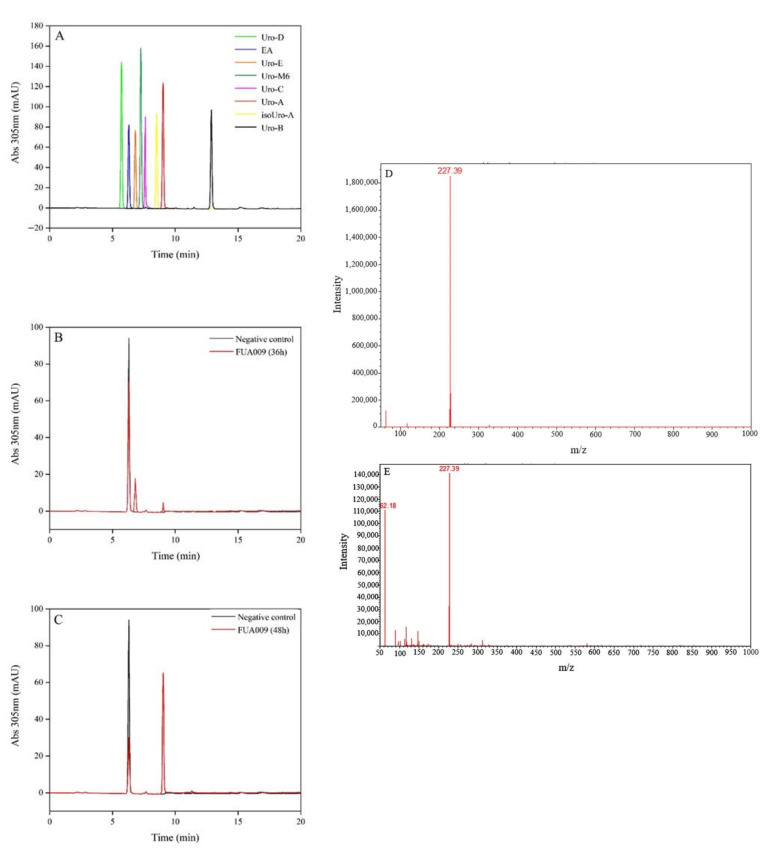
*Lactococcus garvieae* FUA009 could be capable of producing UA from ellagic acid. (**A**) HPLC analysis at 305 nm of the seven urolithin standards. (**B**) HPLC analysis at 305 nm of the fermentation broth of FUA009 at 36 h. (**C**) HPLC analysis at 305 nm of the fermentation broth of FUA009 at 48 h. (**D**) UPLC-MS analysis at 305 nm of the UA standard. (**E**) UPLC-MS analysis at 305 nm of the fermentation product of FUA009 at 48 h. Note: Uro-A—urolithin A, Uro-B—urolithin B, Uro-C—urolithin C, Uro-D—urolithin D, Uro-E—urolithin E, Uro-M6—urolithin-M6, EA—ellagic acid, isoUro-A—isourolithin A.

**Figure 2:**
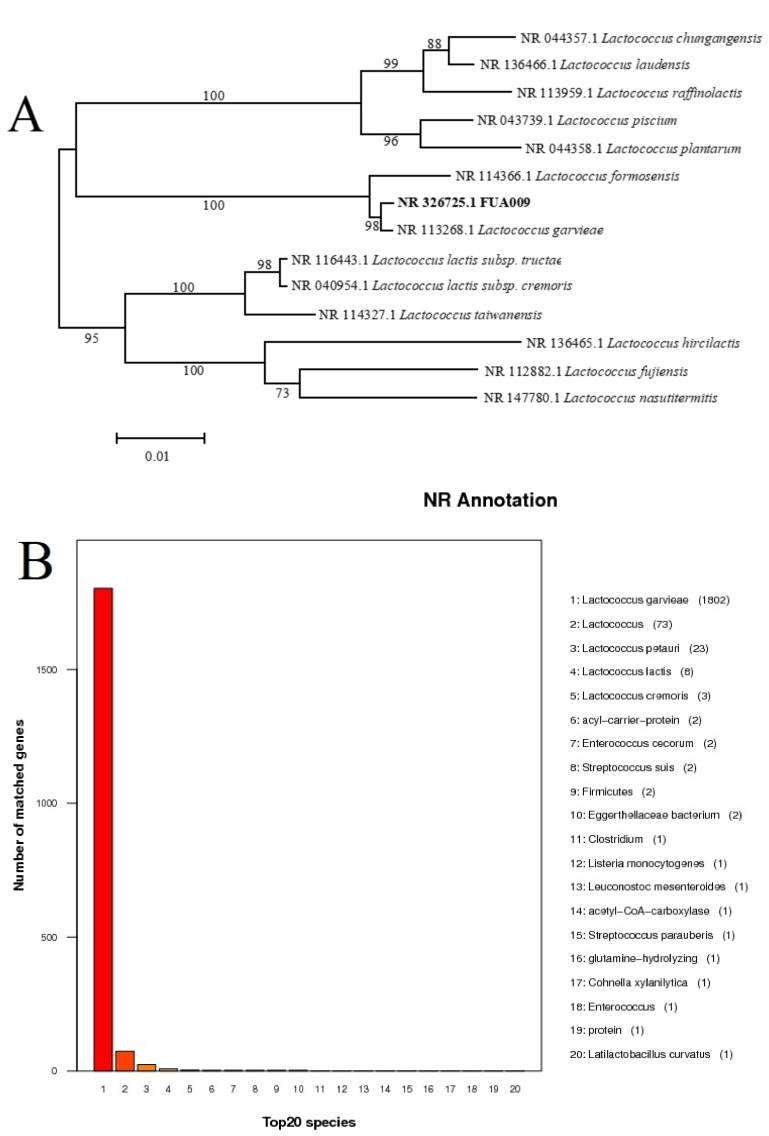
FUA009 was assigned to the species *Lactococcus garvieae*. (**A**) Phylogenetic tree showing the relationships between the *Lactococcus garvieae* FUA009 and other representatives of the family *Lactococcus*. The tree was constructed by using the neighbor-joining method based on 16S rRNA gene sequences. The distance matrix was calculated using the Jukes and Cantor method. Bar: 1% nucleotide sequence difference. Numbers at nodes (≥70%) indicate support for internal branches within the tree obtained using bootstrap analysis (percentages of 500 re-samplings). (**B**) The species annotation results of *Lactococcus* *garvieae* FUA009 in the Non-Redundant Protein Database.

More than 50 strains of bacteria were isolated from the feces of the male volunteer. In the effort to find a single bacterium with the activity of metabolizing ellagic acid to produce UA, we inoculated every single bacterium separately in ABB liquid medium containing 1% ellagic acid and fermented under anaerobic conditions at 37 °C for 0 h, 12 h, 24 h, 36 h, and 48 h. HPLC was used to analyze the presence of urolithins in the fermentation broth. The suspected UA could be detected in the fermentation broth after 36 h of fermentation (Figure 1B,C). The result of HPLC showed that the retention time of two substances in the fermentation broth of the bacterium FUA009 at 36 h was similar to that of urolithin E and the UA standard (Figure 1B). However, the suspected urolithin E disappeared at 48 h and the peak area of the suspected UA increased (Figure 1C). Furthermore, ultra-performance liquid chromatography-tandem mass spectrometry (UPLC-MS) was used to determine the molecular weight of the suspected UA produced at 48 h. The result of UPLC-MS confirmed that the molecular weight of this substance was 227.39, which was consistent with UA (Figure 1D,E). The above results demonstrated that the strain FUA009 was a novel intestinal bacterium capable of producing the bioactive metabolite UA from ellagic acid. To identify the UA-producing bacterium FUA009, we performed 16S rRNA gene sequencing analysis, which showed 98% identity to the sequence of the *Lactococcus garvieae* (Figure 2A). The 16S rRNA gene sequence of FUA009 was submitted to GenBank with accession number NR 326725.1. In addition, the results of the whole genome homology further confirmed that FUA009 could be assigned to the species *Lactococcus garvieae* (Figure 2B). The genome size of FUA009 was 2,036,664 bp with an average GC content of 39.72% (Supplementary Figure S1). Plasmid sequences were not detected in the FUA009 genome. A total of 2004 protein-coding genes (CDSs) and 77 RNA-coding genes were predicted. Among the 77 RNA genes, 1 coded for sRNA, 16 coded for rRNAs, and 60 could account for tRNAs.

### 3.2. Evaluation Safety of Lactococcus garvieae FUA009

#### 3.2.1. Antibiotic Resistance Gene Analysis in the Genome and In Vitro

To explore the drug resistance of *Lactococcus* *garvieae* FUA009, the bacteriostatic circle of FUA009 to 27 antimicrobial agents was determined using the KB disk diffusion technique. As shown in Table 1, the results showed that FUA009 was resistant to seven types of antibiotics, namely, norfloxacin, tetracycline, oxacillin, aztreonam, co-trimoxazole, polymyxin B, and clindamycin. Furthermore, 12 genes associated with antibiotic resistance in the FUA009 genome were also identified using the ARDB database (Table 2). In addition, eight putative mobile genetic elements (seven incomplete prophage-related fragments and one possible genomic island) were found in the FUA009 genome (Supplementary Figure S2 and Table S1), but these mobile genetic elements were not adjacent to antibiotic-related genes. The results of the in vitro drug susceptibility tests and the analysis of the above resistance genes in the genome showed that FUA009 was safe in terms of antibiotic resistance.

#### 3.2.2. Hemolysis Assay of *Lactococcus* *garvieae* FUA009

**Figure 3:**
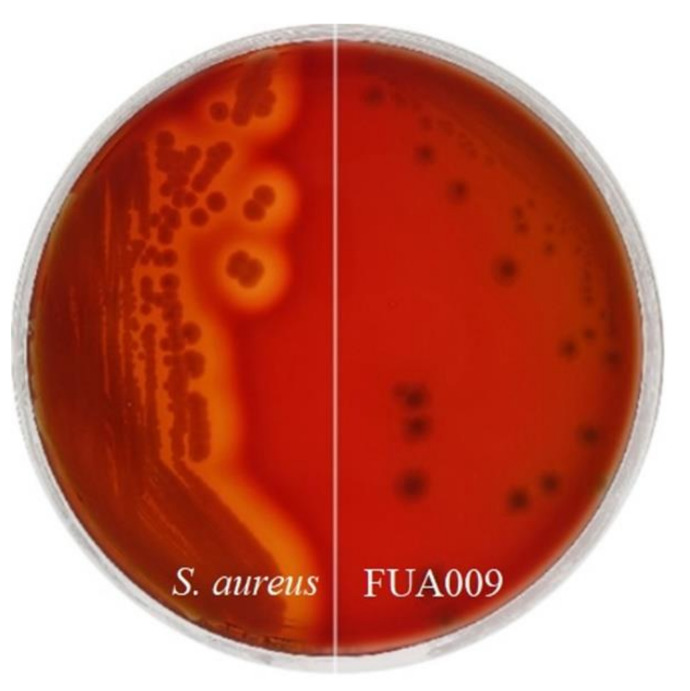
*Lactococcus garvieae* FUA009 did not show any hemolysis ability on the blood agar plates.

The ability of bacteria to lyse erythrocytes was phenotyped by streaking on blood agar plates and observing the level of lysis of erythrocytes. The hemolytic activity of FUA009 was assessed using blood agar containing 6% sheep blood, with *Staphylococcus aureus* as a positive control. As shown in Figure 3, after incubation on blood agar at 37 °C for 24 h, *Lactococcus* *garvieae* FUA009 did not show any hemolysis ability compared with *Staphylococcus aureus*, which produced strong *β*-hemolysis. These results indicated that hemolytic activity was not a concern for the *Lactococcus garvieae* FUA009 applications.

#### 3.2.3. Safety-Related Gene Evaluation in the Genome of FUA009

The potential virulence-related genes of *Lactococcus garvieae* strain FUA009 were identified using the VFDB database. As shown in Table 3, there were 17 putative virulence factor genes (identity > 60%), and these genes might be involved in adherence, immune modulation, exoenzyme, stress survival, and regulation.

### 3.3. Assessment of Probiotic Properties of Lactococcus garvieae FUA009

#### 3.3.1. Evaluation of Stress-Responsive Protein Genes in the *Lactococcus* *garvieae* FUA009 Genome

Long-term survival and colonization in the gastrointestinal tract are the unique features of probiotics. In order to explore whether FUA009 had potential as a probiotic candidate, we analyzed the tolerance protein genes in the FUA009 genome. As shown in Table 4, at least 19 genes in the FUA009 genome encoded proteins related to stress tolerance. The presence of these genes was expected to ensure that FUA009 was tolerant to various environmental stresses, such as acid, bile salts, extreme temperature, metal, and oxidative stress, when it is used as a probiotic candidate.

#### 3.3.2. Evaluation of Adhesion-Related Genes in *Lactococcus* *garvieae* FUA009 Genome

The cell adhesion ability of probiotics is a criterion for evaluating their probiotic properties [24]. To further demonstrate that FUA009 was a promising probiotic candidate for UA production, we searched for annotated gene data related to cell adhesion. As shown in Table 5, seven adhesion-related genes existed in the genome of FUA009, namely, segregation and condensation protein, flagellar hook-associated protein, laminin domain, collagen-binding domain, sortase, and s-ribosylhomocysteine lyase, and these genes might confer good intestinal potential for adhesion to FUA009.

#### 3.3.3. Tolerance of *Lactococcus* *garvieae* FUA009 to Acid and Bile In Vitro

**Figure 4:**
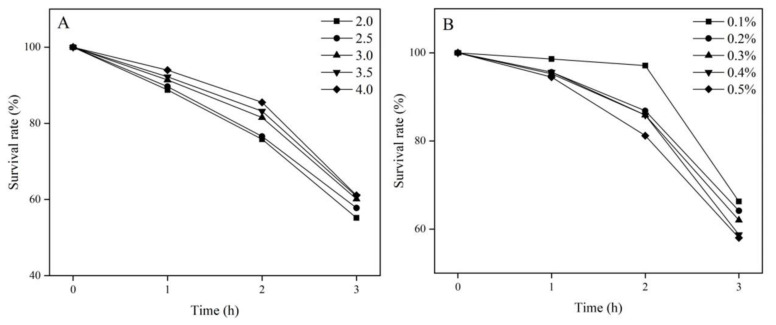
Tolerance of *Lactococcus garvieae* FUA009 in vitro at different pHs (**A**) and concentrations of bile salts (**B**).

Bacteria reach the gastrointestinal tract and act as probiotics only after withstanding the stomach acid barrier. Given that the pH of the stomach generally remains at 2.5–3.5, we examined the survival rates of FUA009 in simulated gastric juice in vitro at pH 2.0–4.0. As shown in Figure 4A, with the decrease in pH and the prolongation of the culture time, the survival rate of the strains decreased to different degrees. However, the cell survival rates of FUA009 were more than 55% after 3 h of treatment. In addition, we analyzed the tolerance of FUA009 to bile salts because the bile salts in the small intestine constitute another barrier for probiotics. The cell survival rates of FUA009 in vitro under the effects of different bile salts are shown in Figure 4B. The survival rate of *Lactococcus* *garvieae* FUA009 was less than 60% after culturing in a 0.4% or 0.5% bile salt medium for 3 h, while the survival rates in 0.3%, 0.2%, and 0.1% bile salt media were 62%, 64%, and 66%, respectively. These phenotypic results showed that the tolerance of FUA009 to acid and bile might be attributed to the presence of tolerance-related genes in its genome.

## 4. Discussion

Ellagic acid, which is a dietary polyphenol that is beneficial to human health, is fairly limited in its bioavailability, and it was established that ellagic acid reaches the colon, where it is metabolized by certain gut microbiota to produce urolithins [25,26,27]. Many studies indicated that urolithins, which are the metabolites of ellagic acid, are the actual bioactive small molecules with antioxidant, anti-inflammatory, and anticancer effects [28,29,30]. UA is one of the end products of ellagic acid metabolism in the intestine tract, which is being paid more and more attention by researchers. To further understand the impact of intestinal microbiota on UA production, it is necessary to isolate an intestinal bacterium with the ability to convert ellagic acid into UA. In the present study, an intestinal bacterium *Lactococcus* *garvieae* FUA009, which is capable of converting ellagic acid to UA, was isolated from the feces of a 26-year-old male volunteer. The results of the 16S rRNA gene sequences and genome sequence indicated that this bacterium belonged to *Lactococcus garvieae*. In addition, we also explored the safety and probiotic characteristics of *Lactococcus* *garvieae* FUA009 based on the complete genome and phenotype analysis.

There is accumulating evidence indicating that the metabolites of dietary polyphenols by intestinal microbiota are closely related to human health [31]. Dietary polyphenols, such as flavonoids and lignans, are macromolecular substances with poor bioavailability [32,33]. Unabsorbed polyphenols are metabolized by certain gut bacteria into better-absorbed small molecule metabolites [34]. However, there are few reports on gut microbes that could metabolize ellagitannins to produce urolithins, especially UA. At present, only four bacteria have been reported, *Gordonibacter* (*G. urolithinfaciens* and *G. pamelaeae*), *Ellagibacter* (*E. isourolithinifaciens*), and *Bifidobacter* (*Bifidobacterium pseudocatenulatum* INIA P815) that can metabolize ellagic acid to urolithins. However, their metabolites are not just urolithin A but also other urolithins metabolized by ellagic acid, such as urolithin B and isourolithin A. In the present study, UA was the only end product of the catabolism of ellagic acid by *Lactococcus* *garvieae* FUA009 (Figure 1). Ellagitannins can be hydrolyzed to hexahydroxydiphenyl acid (HHDP), which is then spontaneously converted to water-insoluble ellagic acid in the gastrointestinal tract [14]. Then, the fecal microbiota utilizes ellagic acid to produce bioactive metabolite urolithins, including urolithin M5, urolithin D, urolithin E, urolithin M6, urolithin C, urolithin M7, UA, isourolithin A, and urolithin B [35]. In our study, given the presence of urolithin E during the fermentation of FUA009, we speculated that FUA009 metabolized EA to produce urolithin M5, urolithin M5 was dehydroxylated to produce urolithin E, and urolithin E was dehydroxylated twice consecutively to produce UA. To date, the enzymes involved in ellagic acid metabolism are not clear, but these enzymes may be lactonase, decarboxylase, and dehydroxylase. The results annotated in the Swiss-Prot database indicated that a phenolic acid decarboxylase (GM_001266) was present in the FUA009 genome. However, the detailed metabolic process of ellagic acid in *Lactococcus* *garvieae* FUA009 needs further study.

Intestinal bacteria that metabolize ellagic acid to produce urolithin A not only contribute to improving the bioavailability of ellagic acid but also have the potential to be considered novel probiotics. Some *Lactococcus* *garvieae* strains isolated from dairy products are thought to be related to the ripening process of dairy products or the organoleptic properties of some artisan cheeses [36]. Given many reports that *Lactococcus garvieae* was the fish pathogen [37], we first evaluated the safety of *Lactococcus garvieae* FUA009 through safety tests and the discovery of safety-related genes. Our results indicated that FUA009 was sensitive to 19 antibiotics and showed no hemolysin activity (Table 1 and Figure 3). Meanwhile, after comparing with ARDB and VFDB databases, 12 antibiotic resistance genes and 17 putative virulence factors were found (Table 2 and Table 3). The horizontal transfer of resistance genes is one of the most important crises facing the medical community up to the present [38]. Plasmids, genomic islands, prophages, and other mobile genetic elements (insertion sequences, transposons, and integrative and conjugative elements) contribute to the spread of antibiotic resistance. There were seven incomplete prophage-related fragments and one possible genomic island in the genome of FUA009. However, the upstream and downstream of the safety-related genes (antibiotics resistance genes and virulence factors genes) in the genome FUA009 did not contain mobile elements, such as gene islands, indicating that these safety-related genes were less likely to be transferred. Knowledge about the virulence determinants of *Lactococcus* *garvieae* is still limited, and most related studies focused on clinical isolates from fish, which demonstrated that virulence factors are primarily related to hemolytic activity, capsule formation, and siderophore production [39]. In *Lactococcus* *garvieae*, the encoding genes *cpsC*, *cpsD*, *cpsE*, *cpsG*, *cpsI*, *cpsJ*, and *cpsK* are necessary for the formation of the capsule [40]. However, these genes were not found in the FUA009 using genomic analysis. The FUA009 genome was also absent of the genes encoding for iron uptake (*fepB*, *fepC*, *fepD*, *fecB*, *fecC*, *fecD*, *feoA*, and *feoB*). These results further indicated that *Lactococcus* *garvieae* FUA009 was safe, and the ability of FUA009 to metabolize ellagic acid to produce UA made it promising for commercial application.

Due to the positive effects (cardiovascular protective, anti-inflammatory, and anticancer properties) of urolithin A on health, the screening and identification of urolithin A-producing bacteria have become a research hotspot, and such bacteria have the potential to become novel probiotics. As a lactic acid bacterium, the probiotic properties of *Lactococcus garvieae* have been studied [41,42]. In this study, *Lactococcus garvieae* FUA009 was less tolerant to acid and bile salts than some probiotics, such as *Lactobacillus* GG, that have been commercially applied [43]. However, *Lactococcus garvieae* FUA009 also exhibited superior probiotic properties (Figure 4) compared with all the reported *Lactococcus garvieae* and some probiotics [44,45]. In addition, we evaluated the cell viability of *Lactococcus garvieae* FUA009 after an acid transit (pH 2.0 and pH 3.0) and the residual cells were treated with 0.3% and 0.5% bile salts (pH 7.0), respectively. The results indicated that the cell viability of *Lactococcus garvieae* FUA009 decreased significantly after treatment with acid (pH 2.0) and 0.5% bile salt in turn (Supplementary Figure S3). However, 0.3% bile salt is often used to screen probiotics because the concentration of bile salt in the human intestine can be as high as 0.3% [46,47]. After the acid (pH 2.0 and pH 3.0) treatment, the residual cells of *Lactococcus garvieae* FUA009 were exposed to 0.3% bile salt and the final cell viabilities were 41% and 48%, respectively (Supplementary Figure S3). These characteristics ensure that probiotics can be transported to their niche and then have a health-promoting effect. The F_0_F_1_-ATPase is recognized as the major regulator of intracellular pH, and eight proteins (GM_000429 to GM_000436) were found in the FUA009 genome (Table 4). Moreover, in addition to divalent metal cation transporter MntH; the cadmium-, zinc-, and cobalt-transporting ATPase; and a citrate-sodium symporter, we also found three copy cation transport protein genes, four copy CorA-like Mg^2+^ transporter genes, and three copy potassium/sodium uptake protein genes in the FUA009 genome. These genes suggested that the *Lactococcus garvieae* FUA009 might be tolerant to the ionic concentration variation. Similar results of 23 proteins that participated in cation transport were explored in *Lactobacillus plantarum* 5-2 [48]. The adhesion of probiotics to the intestinal mucosa and epithelial cells facilitates their colonization and contributes to the healthy effects of probiotics [24]. We also found eight adhesion-related genes, such as the genes that annotated the segregation and condensation protein A and B (scpA and scpB), the flagellar hook-associated protein, laminin, the collagen-binding domain, sortase, and s-ribosylhomocysteine lyase (Table 5). The same results were also found in the commercially available probiotics *Bacillus coagulans* S-lac and *Bacillus coagulans* GBI-30 [24]. Based on the above, the UA-producing bacterium *Lactococcus* *garvieae* FUA009 has the potential to become a novel probiotic candidate.

## 5. Conclusions

In this study, we isolated a bacterium *Lactococcus* *garvieae* FUA009, which was capable of metabolizing ellagic acid to produce UA, from the feces of a healthy volunteer. The results of genome screens and phenotypic analysis indicated that FUA009 had apparent safety and probiotic characteristics. Therefore, given that urolithin A has various bioactivities beneficial to health, *Lactococcus* *garvieae* FUA009 is expected to be further applied in the development of functional foods and health products. However, the metabolic pathways of FUA009 using ellagic acid as the substrate to produce UA require further study.

## Figures and Tables

**Table 1:** Drug sensitivity test results of *Lactococcus garvieae* FUA009 to 27 antibiotics.

Antibiotic	Drug Contents (μg)	Standard for Judging Diameter of Inhibition Zone Diam (mm) [22]			Zone Diam (mm)	Antibacterial Effect
		Resistant (R)	Intermediate (I)	Susceptible (S)		
Amikacin	30	≤14	15~16	≥17	19 ± 0.5	S
Norfloxacin	10	≤12	13~16	≥17	12 ± 0.6	R
Ofloxacin	5	≤12	13~15	≥16	20 ± 0.3	S
Ciprofloxacin	5	≤15	16~20	≥21	17 ± 0.2	I
Levofloxacin	5	≤12	13~16	≥17	20 ± 0.1	S
Erythromycin	15	≤13	14~22	≥23	26 ± 0.6	S
Tetracycline	30	≤14	15~18	≥19	8 ± 0.3	R
Cefuroxime	30	≤14	15~17	≥18	35 ± 0.7	S
Cefazolin	30	≤14	-	≥15	31 ± 0.3	S
Cefalotin	30	≤14	15~17	≥18	25 ± 0.6	S
Cefotaxime	30	≤22	23~25	≥26	36 ± 0.2	S
Cefatriaxone	30	≤13	14~20	≥21	32 ± 0.2	S
Ceftazidime	30	≤14	15~17	≥18	30 ± 0.6	S
Piperazoline	100	≤28	-	≥29	31 ± 0.2	S
Ampicillin	10	≤16	18~24	≥25	29 ± 0.6	S
Oxacillin	1	≤17	-	≥25	17 ± 0.1	R
Penicillin G	10	≤28	-	≥29	33 ± 0.3	S
Aztreonam	30	≤15	16~21	≥22	0	R
Co-trimoxazole	23.75	≤10	11~15	≥16	0	R
Furadantin	300	≤14	15~16	≥17	23 ± 0.6	S
Chloramphenicol	30	≤12	13~17	≥18	26 ± 0.6	S
BacillosporinB	300	≤11	12~14	≥15	0	R
Clindamycin	2	≤13	14~17	≥18	0	R
Kanamycin	30	≤12	13~14	≥15	21 ± 0.2	S
Gentamicin	10	≤12	13~14	≥15	17 ± 0.3	S
Streptomycin	10	≤11	12~14	≥15	17 ± 0.5	S
Vancomycin	30	≤14	15~16	≥17	18 ± 0.2	S

**Table 2:** Putative antibiotic resistance genes identified in the genome of *Lactococcus garvieae* FUA009.

Resistance Type	Antibiotic Resistance	Identity (%)	Gene Locus
Tets	Tetracycline	100	GM_000049
Vanra	Vancomycin, teicoplanin	42.4	GM_000289
Pmra	Ciprofloxacin, norfloxacin	50.0	GM_000376
Mdr	-	41.2	GM_000399
Pbp2x	Penicillin	42.7	GM_000615
Vanra	Vancomycin, teicoplanin	43.7	GM_000618
Tet38	Tetracycline	42.8	GM_000769
Lsa	Lincosamide, streptogramin_b,Macrolide	53.5	GM_000802
Emea	Fluoroquinolone	82.0	GM_000980
Baca	Bacitracin	56.9	GM_001070
Vanrg	Vancomycin	46.4	GM_001619
Vanz	Teicoplanin	45.5	GM_001828

**Table 3:** Putative virulence factors in the *Lactococcus garvieae* FUA009 genome.

Role	Virulence Factor	Related Genes	Identity (%)	Gene Locus
Adherence	Streptococcal plasmin receptor/GAPDH	*plr/gapA*	84.8	GM_001975
	EF-Tu	*tuf*	72.6	GM_001667
	GroEL	*groEL*	69.8	GM_000286
	Fibronectin-binding proteins	*pavA*	63.3	GM_000679
Immune modulation	Capsule	*rmlC*	89.1	GM_000129
	Capsule	*rmlA*	88.9	GM_000128
	Capsule	*STER_1222*	85.6	GM_000131
	Capsule	*hasC*	78.6	GM_000658
	Capsule	*rmlD*	71.2	GM_000132
	Capsule	*gnd*	68.2	GM_001520
	Capsule	*STER_1434*	62.9	GM_000136
Exoenzyme	Streptococcal enolase	*eno*	92.0	GM_001501
	Hyaluronidase	*EF0818*	60.8	GM_001436
Stress survival	Trigger factor	*tig/ropA*	67.2	GM_000359
	ClpP	*clpP*	64.2	GM_000379
	ClpE	*clpE*	62.5	GM_001645
Regulation	LisR/LisK	*lisR*	64.1	GM_000453

**Table 4:** Stress-responsive proteins of *Lactococcus garvieae* FUA009 in the whole genome.

Type of Stress Response	Protein	Related Genes	Gene Locus
Acid stress response	F_0_F_1_-ATPase	*atpA*, *atpB*, *atpC*, *atpD*, *atpE*, *atpF*, *atpG*, *atpH*	GM_000429, GM_000430, GM_000431, GM_000432, GM_000433, GM_000434, GM_000435, GM_000436
	Na^+^/H^+^ antiporter family	-	GM_001303
Bile salts stress response	Cyclopropane-fatty-acyl-phospholipid synthase	*cfa*	GM_000663
	ABC transporter ATP-binding protein YxdL	*yxdL*	GM_000471
	Sodium/hydrogen exchanger family	-	GM_000272, GM_000959, GM_000988, GM_001719
Temperature stress response	Heat shock protein 9/12	-	GM_000598
	Cold shock protein	*cspA*	GM_000105, GM_000573, GM_001587, GM_001590
Metal stress response	Divalent metal cation transporter MntH	*mntH*	GM_000991
	Cation transport protein	-	GM_000063, GM_001371, GM_001582
	CorA-like Mg^2+^ transporter protein	-	GM_001262, GM_001495, GM_001671, GM_001771
	Cadmium, zinc and cobalt-transporting ATPase	*cadA*	GM_000577
	Potassium/sodium uptake protein	*ntpJ*	GM_001371, GM_001582, GM_000063
	Citrate-sodium symporter	*citP*	GM_001096
Oxidative stress response	Alkyl hydroperoxide reductase	*ahpC*	GM_001062
	Glutathione peroxidase	*gpo*	GM_000931
	Thioredoxin reductases	*trxB*	GM_000780
	Superoxide dismutase [Fe]	*sodA*	GM_000304

**Table 5:** Cell-adhesion-related proteins in the *Lactococcus garvieae* FUA009 genome.

Protein/Domain	Related Genes	Gene Locus
Segregation and condensation protein B	*scpB*	GM_001136
Segregation and condensation protein A	*scpA*	GM_001137
Flagellar hook-associated protein	*flgK, flgL*	GM_000659, GM_000034
Laminin domain II	-	GM_001909
Collagen binding domain	-	GM_001621
Sortase A	*strA*	GM_000696
S-ribosylhomocysteine lyase	*luxS*	GM_001854

## Data Availability

The data presented in this study are available on request from the corresponding author.

## Supplementary Materials

The following supporting information can be downloaded at: https://www.mdpi.com/article/10.3390/foods11172621/s1, Figure S1: Circos plot for assembled genome of *Lactococcus garvieae* strain FUA009.; Figure S2: The genomic island in the FUA009 genome; Figure S3: Cell viability of *Lactococcus garvieae* FUA009 cultured in ABB medium at pH 2.0 and pH 3.0, followed by ABB medium containing 0.3% bile salt (A) or 0.5% bile salt (B) at pH 7.0; Table S1: Putative prophage fragment in FUA009 genome.
