# Supplementary material for: Lactococcus garvieae FUA009, a Novel Intestinal Bacterium Capable of Producing the Bioactive Metabolite Urolithin A from Ellagic Acid
Source: Foods. 2022 Aug 29;11(17):2621. doi: 10.3390/foods11172621 (PMC9455165; doi:10.3390/foods11172621)
Supplement: Supplementary file 1 [file foods-11-02621-s001.zip › foods-1831329-supplementary.pdf]

Supplementary material

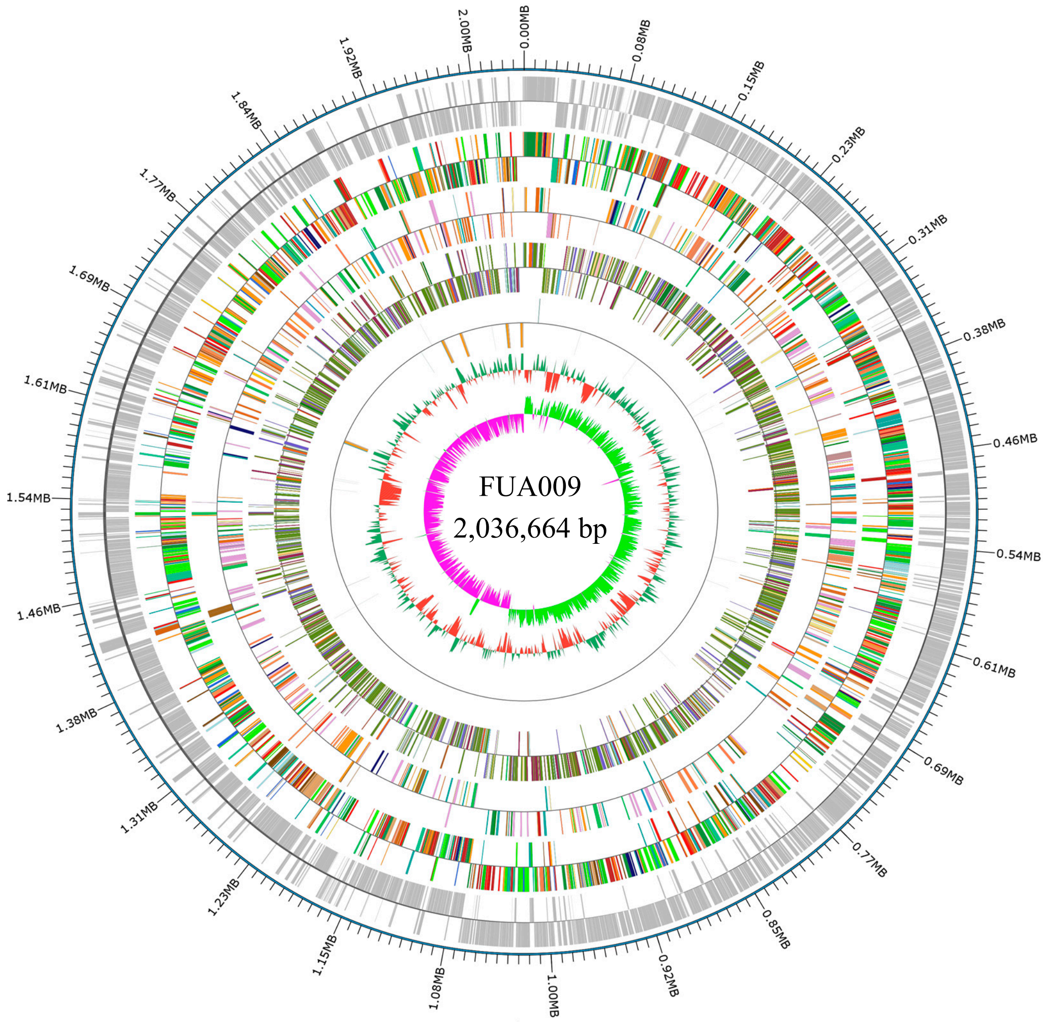

Supplementary Figure S1. Circos plot for assembled genome of *Lactococcus garvieae* strain FUA009. The circles (outer to inner) represented coding genes, gene function annotation results, ncRNA, genome GC content and genome GC skew value, respectively. Circles 1 and 2 were coding genes. The gene function annotation results were COG (circle 3 and 4), KEGG (circle 5 and 6) and GO (circle 7 and 8). The circle 9 and 10 represented ncRNA of genome. The genome GC content (circle 11 and 12): the inward red part indicated that the GC content in this region was lower than the average GC content in the whole genome, and the outward green part was opposite. The circle 13 and 14 represented genome GC skew value, the inward pink part indicated that the content of G in the region was lower than that of C, and the outward light green part was opposite.

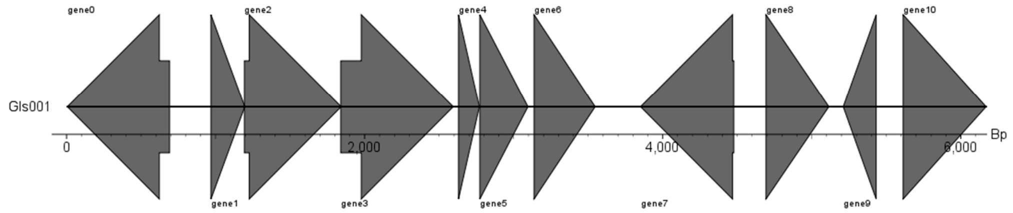

Supplementary Figure S2. The genomic island in the FUA009 genome.

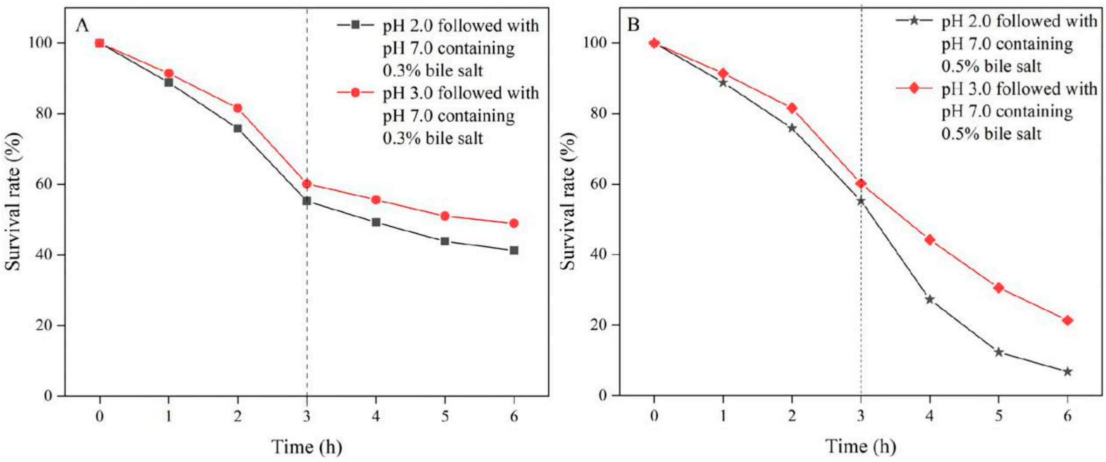

Supplementary Figure S3. Cell viability of *Lactococcus garvieae* FUA009 cultured in ABB medium at pH 2.0 and pH 3.0, followed by ABB medium containing 0.3% bile salt (A) or 0.5% bile salt (B) at pH 7.0.

Table S1. Putative prophage fragment in FUA009 genome.

Prophage_ID	Locate	Length	GC%	Gene Locus
Prophage_1	Chr1	26695	38.06	GM_000062,GM_000063,GM_000064,GM_000065,GM_000066,GM_000067,GM_000068,GM_000069,GM_000070,GM_000071,GM_000072,GM_000073,GM_000074,GM_000075,GM_000076,GM_000077,GM_000078,GM_000079,GM_000080,GM_000081,GM_000082,GM_000083,GM_000084,GM_000085,GM_000086,GM_000087,GM_000088
Prophage_2	Chr1	31145	39.41	GM_000160,GM_000161,GM_000162,GM_000163,GM_000164,GM_000165,GM_000166,GM_000167,GM_000168,GM_000169,GM_000170,GM_000171,GM_000172,GM_000173,GM_000174,GM_000175,GM_000176,GM_000177,GM_000178,GM_000179,GM_000180,GM_000181,GM_000182,GM_000183,GM_000184,GM_000185,GM_000186,GM_000187,GM_000188,GM_000189,GM_000190,GM_000191,GM_000192,GM_000193,GM_000194,GM_000195,GM_000196,GM_000197,GM_000198,GM_000199,GM_000200,GM_000201,GM_000202,GM_000203,GM_000204
Prophage_3	Chr1	64931	39.94	GM_000315,GM_000316,GM_000317,GM_000318,GM_000319,GM_000320,GM_000321,GM_000322,GM_000323,GM_000324,GM_000325,GM_000326,GM_000327,GM_000328,GM_000329,GM_000330,GM_000331,GM_000332,GM_000333,GM_000334,GM_000335,GM_000336,GM_000337,GM_000338,GM_000339,GM_000340,GM_000341,GM_000342,GM_000343,GM_000344,GM_000345,GM_000346,GM_000347,GM_000348,GM_000349,GM_000350,GM_000351,GM_000352,GM_000353,GM_000354,GM_000355,GM_000356,GM_000357,GM_000358,GM_000359,GM_000360,GM_000361,GM_000362,GM_000363,GM_000364,GM_000365,GM_000366,GM_000367,GM_000368,GM_000369,GM_000370,GM_000371,GM_000372,GM_000373,GM_000374,GM_000375
Prophage_4	Chr1	44737	35.19	GM_000717,GM_000718,GM_000719,GM_000720,GM_000721,GM_000722,GM_000723,GM_000724,GM_000725,GM_000726,GM_000727,GM_000728,GM_000729,GM_000730,GM_000731,GM_

				000732,GM_000733,GM_000734,GM_000735,GM_000736,GM_000737,GM_000738,GM_000739,GM_000740,GM_000741,GM_000742,GM_000743,GM_000744,GM_000745,GM_000746,GM_000747,GM_000748,GM_000749,GM_000750,GM_000751,GM_000752,GM_000753,GM_000754,GM_000755,GM_000756,GM_000757,GM_000758,GM_000759,GM_000760,GM_000761,GM_000762,GM_000763,GM_000764,GM_000765,GM_000766,GM_000767
Prophage_5	Chr1	63088	38.18	GM_001109,GM_001110,GM_001111,GM_001112,GM_001113,GM_001114,GM_001115,GM_001116,GM_001117,GM_001118,GM_001119,GM_001120,GM_001121,GM_001122,GM_001123,GM_001124,GM_001125,GM_001126,GM_001127,GM_001128,GM_001129,GM_001130,GM_001131,GM_001132,GM_001133,GM_001134,GM_001135,GM_001136,GM_001137,GM_001138,GM_001139,GM_001140,GM_001141,GM_001142,GM_001143,GM_001144,GM_001145,GM_001146,GM_001147,GM_001148,GM_001149,GM_001150,GM_001151,GM_001152,GM_001153,GM_001154,GM_001155,GM_001156,GM_001157,GM_001158,GM_001159,GM_001160,GM_001161,GM_001162,GM_001163,GM_001164,GM_001165,GM_001166,GM_001167,GM_001168,GM_001169,GM_001170,GM_001171,GM_001172,GM_001173,GM_001174,GM_001175,GM_001176,GM_001177,GM_001178,GM_001179,GM_001180,GM_001181,GM_001182,GM_001183,GM_001184,GM_001185,GM_001186,GM_001187,GM_001188,GM_001189,GM_001190,GM_001191
Prophage_6	Chr1	132392	37.24	GM_001451,GM_001452,GM_001453,GM_001454,GM_001455,GM_001456,GM_001457,GM_001458,GM_001459,GM_001460,GM_001461,GM_001462,GM_001463,GM_001464,GM_001465,GM_001466,GM_001467,GM_001468,GM_001469,GM_001470,GM_001471,GM_001472,GM_001473,GM_001474,GM_001475,GM_001476,GM_001477,GM_001478,GM_001479,GM_001480,GM_001481,GM_001482,GM_001483,GM_001484,GM_001485,GM_001486,GM_001487,GM_001488,GM_001489,GM_001490,GM_001491,GM_001492,GM_001493,GM_001494,GM_001495,GM_001496,GM_001497,GM_001498,GM_001499,GM_001500,GM_001501,GM_001502,GM_001503,GM_001504,GM_001505,GM_001506,GM_001507,GM_001508,GM_001509,GM_001510,GM_001511,GM_001512,GM_001513,GM_001514,GM_001515,GM_001516,GM_001517,GM_001518,GM_001519,GM_001520,GM_001521,GM_001522,GM_001523,GM_001524,GM_001525,GM_001526,GM_001527,GM_001528,GM_001529,GM_001530,GM_001531,GM_001532,GM_001533,GM_001534,GM_001535,GM_001536,GM_001537,GM_001538,GM_001539,GM_001540,GM_001541,GM_001542,GM_001543,GM_001544,GM_001545,GM_001546,GM_001547,GM_001548,GM_001549,GM_001550,GM_001551,GM_001552,GM_001553,GM_001554,GM_001555,GM_001556,GM_001557,GM_001558,GM_001559,GM_001560,GM_001561,GM_001562,GM_001563,GM_001564,GM_001565,GM_001566,GM_001567,GM_001568,GM_001569,GM_001570,GM_001571,GM_001572,GM_001573,GM_001574,GM_001575,GM_001576,GM_001577,GM_001578,GM_001579,GM_001580,GM_001581,GM_001582,GM_001583,GM_001584,GM_001585,GM_001586,GM_001587,GM_001588,GM_001589,GM_001590,GM_001591,GM_001592,GM_001593,GM_001594
Prophage_7	Chr1	34427	39.76	GM_001914,GM_001915,GM_001916,GM_001917,GM_001918,GM_001919,GM_001920,GM_001921,GM_001922,GM_001923,GM_001924,GM_001925,GM_001926,GM_001927,GM_001928,GM_001929,GM_001930,GM_001931,GM_001932,GM_001933,GM_001934,GM_001935,GM_001936,GM_001937,GM_001938,GM_001939,GM_001940,GM_001941,GM_001942,GM_001943,GM_001944,GM_001945,GM_001946,GM_001947,GM_001948,GM_001949,GM_001950,GM_001951,GM_001952
